# A Preclinical Model of Human Liver Using Precision Cut Tissue Slice Culture

**DOI:** 10.12688/f1000research.162495.1

**Published:** 2025-06-10

**Authors:** Owen McGreevy, Timothy Gilbert, Maria-Danae Jessel, Mohammed Bosakhar, Stephen Fenwick, Hassan Malik, Christopher Goldring, Laura Randle

**Affiliations:** 1 University of Liverpool Department of Pharmacology and Therapeutics, Liverpool, England, UK; 2 Equal Contribution, Liverpool, UK; 3 Hepatobiliary Surgery, The Royal Liverpool University Hospital, Liverpool, UK

**Keywords:** CELLBLOKS®, ex-vivo, human precision cut tissue slices (hPCTS), Millicell inserts, organotypic culture, polyethylene terephthalate (PET) membrane, polytetrafluoroethylene (PTFE) membrane, preclinical model

## Abstract

Preclinical models vary in complexity and cost. Traditional 2D cell cultures that are high throughput and cost effective, but lack the complexity of multicellular interactions. Animal models and more complex, but are costly, raise ethical concerns and are not a human model to better understand human disease or response to novel treatments. Human precision cut tissue slice (hPCTS) models bridge this gap, maintaining the architecture and microenvironment of original tissues.

This study examines the viability and functionality of hPCTS using different tissue culture formats. Previous studies have cultured hPCTS with gentle agitation, either on an insert or floating in tissue culture medium. More recently the use of a proprietary flow system for hPCTS culture has been explored, aiming to provide a more physiologically relevant environment. CELLBLOKS® provide a commercially available flow system platform designed for cell culture that we adapted to accommodate hPCTS. hPCTS were cultured for 15 days using an organotypic polytetrafluoroethylene Millicell insert, a CELLBLOKS® hydrophilic polyethylene terephthalate flow system plate or without an insert. Viability was assessed through MTS assays, while functionality was determined by measuring urea and albumin secretion across the 15 days in culture. The Millicell inserts maintained higher and more consistent viability and functionality over 15 days. Slices cultured with no inserts showed decreased viability and functionality after 7 days in culture. In contrast, CELLBLOKS® cultured hPCTS showed significantly decreased viability and function after 3 days in culture.

This study suggests that while the CELLBLOKS® system shows promise for 2D cell line cultures, Millicell Biopore™ inserts offer a more reliable method for maintaining complex hPCTS cultures, preserving both viability and function. As a viable, human-specific alternative to animal models, hPCTS support the 3Rs and have the potential to reduced and potentially replace the use of animals in preclinical research, improving human disease modelling.

## Introduction

Accurate models that recapitulate human biology are essential for understanding disease mechanisms and testing novel therapies. However, no preclinical model is perfect. Two-dimensional (2D) cell culture is a cost-effective, reproducible method for studying cellular processes but lacks the complexity of native tissue. ^1^ Three-dimensional (3D) organoid cultures improve on this but fail to fully replicate in vivo architecture. Animal models provide a whole-system approach but do not fully mimic human disease. Patient-derived xenograft (PDX) models improve clinical relevance by transplanting human tissue into immunodeficient mice but have limitations such as graft uptake issues, cost, time demands, and mouse-specific evolution limiting clinical relevance. Ethical concerns regarding animal use in research persist. ^2^

Human precision-cut tissue slices (hPCTS) have re-emerged as physiologically relevant models for studying disease biology and testing therapeutics. ^3^ Unlike traditional cell culture, hPCTS retain the tissue’s native architecture and microenvironment. ^4^ This provides researchers with a physiologically representative model to explore various disease processes and a more clinically relevant platform for therapeutic studies. The use of hPCTS has significant potential to reduce and replace animals in preclinical research. Our institution uses up to 150 mice and 30 rats annually to model liver disease and determine drug efficacy/safety, with 12 studies using rodent hepatocytes published since 2017. Following the introduction of hPCTS within our group in 2021, tissue slice models have replaced animals in 12 research projects.

An advantage of hPCTS is their adaptability and multiple tissue compatibility, lung, liver, prostate, and brain. ^5^ De Graaf *et al.* (2010) published a standardised liver and intestinal hPCTS protocol, renewing interest in this technique. ^5^ This was limited by relatively short-term viability of several days due to issues maintaining sufficient oxygenation and nutrition. To extend viability, inserts have been used to help maintain an air liquid interface producing a physiologically relevant oxygen concentration gradient. ^6^ The use of interconnecting culture wells on a rocking platform to simulate a flow system has also been shown to improve PCTS viability and function. ^7^

Enhancing the long-term viability of hPCTS is crucial for their application as preclinical models capable of predicting therapeutic outcomes and reducing reliance on animal testing. Here, we present findings from a pilot study examining hPCTS viability under three culture conditions.

## Methods

### Patient selection and tissue collection

Liver tissue was collected from five patients ( Table 1) undergoing planned liver tumour resections at the Royal Liverpool University Hospital (RLUH). Patients provided written informed consent before sample collection. Ethical approval and written consent were obtained under the NIHR PINCER platform study (NW REC 15/NW/0477), ^8^ and the University of Liverpool Ethics Board adhering to the declaration of Helsinki. ^9, 10^ All demographic data were anonymised. No extra selection criteria were applied. After resection, normal liver tissue was immersed in University of Wisconsin (UW) buffer (Bridge to Life Ltd, #BUWC-1000) and transported to the laboratory within one hour to minimize ischemia. ^5^ Tissue handling complied with the UK Human Tissue Authority (HTA) Act 2004.

### hPCTS generation

**Figure 1.**
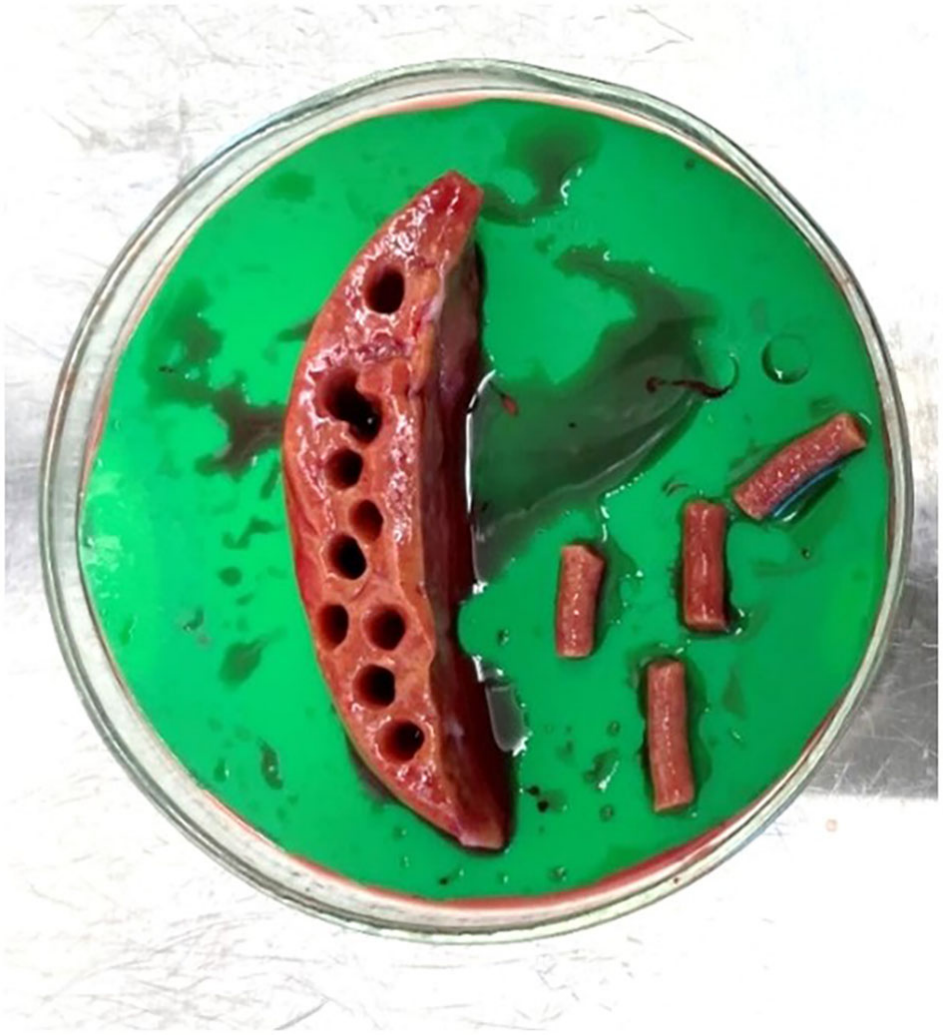
Section of normal liver tissue that has been cored to produce multiple 5 mm diameter cores ready for insertion into the Krumdieck tissue slicer.

Following Kenerson *et al*. ^11^ liver specimens were removed from UW buffer using forceps and placed in a petri dish (Greiner Bio-One, #639102) with sufficient UW buffer (~10 ml) to remain moist. Multiple 5 mm tissue cores were prepared using a coring press (Alabama Research & Development, Model MD5000) ( Figure 1). Cores were sectioned into 250 μm slices using a precision cut tissue slicer (Alabama R&D Tissue Slicer MD6000), (arm speed 4, blade speed 4) ( Figure 3) with 4 ^o^C 1x Krebs Henseleit Buffer (KHB) ( Table 2). Slices were pooled in a shared KHB reservoir, ensuring mixing of slices from different cores, and screened for size consistency, discarding incomplete slices. Prepared slices were transferred into individual wells of a 24 well plate (Cellstar ^®^, Greiner Bio-One, #662160) containing 400 μl of modified Williams E Media (WEM, Gibco, #12551032) and incubated (37 ^o^C, 5% CO _2_) on an orbital shaker (SLS Lab Basics Digital Orbital Shaker, SLS6060) (95 RPM) for 1 hour for ‘slice recovery’ and removal of debris from slicing. ^5, 11^

**Table 1.** Patient demographic and clinical characteristics for the liver specimens used to generate human precision-cut tumour slices (hPCTS). Abbreviations: BMI, body mass index; Ex, ex-smoker; ETOH xs, alcohol excess; Pre-op, preoperative; PVE, portal vein embolization; SACT, systemic anticancer therapy; FOLFOX, 5-fluorouracil + leucovorin + oxaliplatin; FOLFIRI, 5-fluorouracil + leucovorin + irinotecan; CRLM, colorectal liver metastases; IVC, inferior vena cava.

Patient	Age	Sex	BMI	Smoker	Diabetes	ETOH xs	Pre-op jaundice	Pre-op PVE	Neoadjuvant therapy	Operation	Histology		
											Primary	Fibrosis (Ishak score)	Steatosis (grade)
Patient 1	32	Female	27.1	Ex	No	No	No	No	None	Left lateral sectionectomy	Adenoma	No	No
Patient 2	70	Male	21.9	No	No	No	No	No	SACT - FOLFOX	Right posterior sectionectomy	CRLM	No	No
Patient 3	79	Female	24.1	Ex	No	No	No	No	SACT - FOLFOX and right PV ligation	Stage Left lateral sectionectomy	CRLM	No	No
Patient 4	70	Female	18.8	No	Type 2	No	No	Yes	SACT - FOLFOX + Cetuximab	Extended right hepatectomy	CRLM	No	No
Patient 5	67	Female	26.8	No	No	No	No	No	SACT - FOLFIRI + Panitumumab	Right hepatectomy + IVC resection	CRLM	No	Yes (moderate)

**Table 2.** Reagents required for the generation of hPCTS.

Williams E Media (WEM) supplemented			
Solution (A)	Volume	Final	Stock
Nicotinamide	6 ml	12 mM	100 mM
L - Ascorbic Acid	6 ml	175 uM	14.5 mM
Sodium Bicarbonate	13.4 ml	0.225% w/v (26.8 mM)	7.5% 1 M
HEPES	10 ml	20 mM	1 M
Glucose	13.9 ml	27.37 mM	1 M
Sodium Pyruvate	5 ml	1 mM	100 mM
L-Glutamine	5 ml	2 mM	200 mM
Penicillin Streptomycin	2 ml	0.4%	
ITS+ Premix	5 ml	1%	100%
WEM	433.7 ml		

10 x Krebs Henseleit Buffer (KHB) (note: combine reagents in two separate flasks as prompted and combine at the end)				
Chemical	Molecular weight	Amount required/5L Total	10X Stock	Flask
ddH _2_O		2L		A
CaCl _2_.2H _2_O	147.0	18.35 g	25 mM	A
KCl	74.55	18.65 g	50 mM	B
NaCl	58.44	345 g	1180 mM	B
MgSO _4_.7H _2_O	246.48	13.55 g	110 mM	B
KH _2_PO _4_	136.09	8.15 g	120 mM	B
ddH _2_O				B

1 x Krebs Henseleit Buffer (KHB)		
Solution	Volume	Conc
KHB	100 ml	10X
NaHCO _3_	25 ml	1 M
Glucose	25 ml	1 M
HEPES	10 ml	1 M
dH _2_O	840 ml	N/A

### hPCTS culture

**Figure 2.**
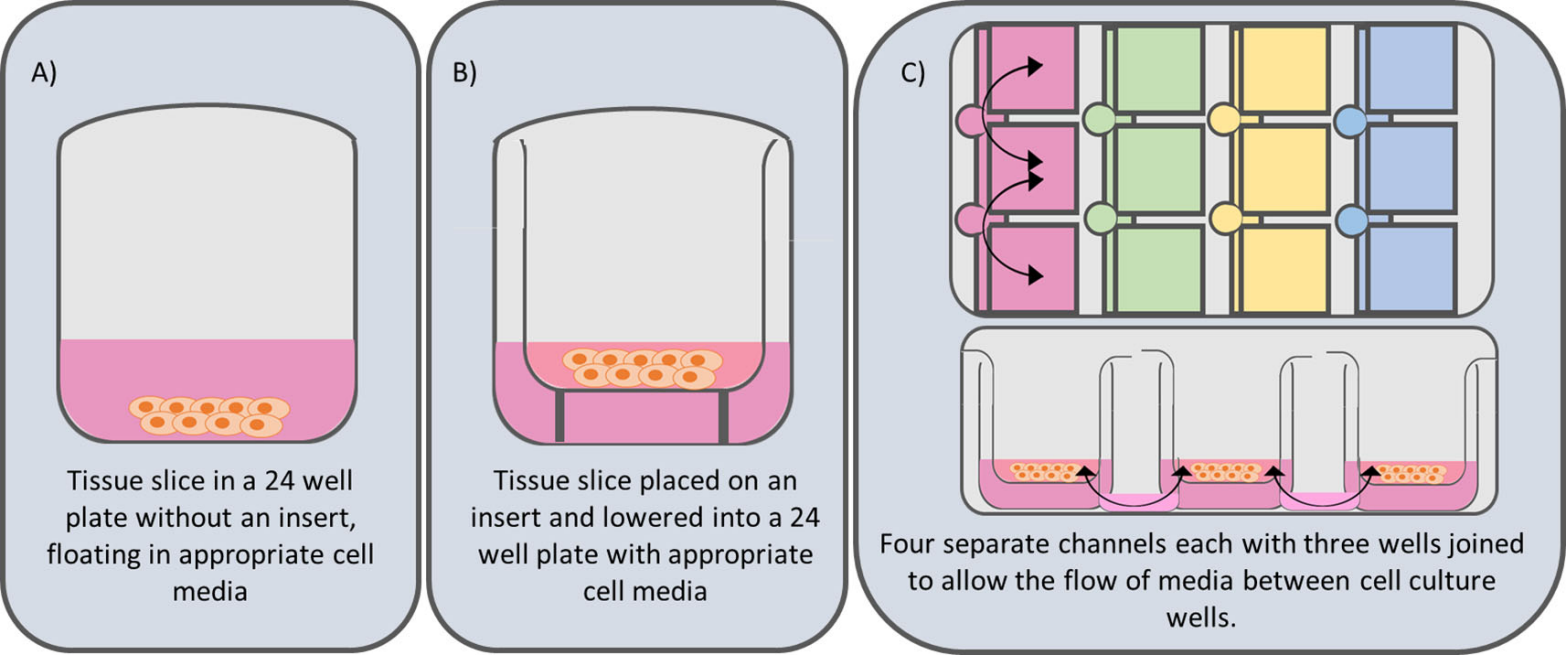
Comparison of 3 culture systems. System A) this is the simplest systems and is derived from that published by de Graf *et al.* ^5^ and consists of a tissue slice submerged in cell culture media in a 24 well culture plate. System B) Involves the addition of a Millicell Standing Cell-Culture insert (Merek PICM01250-12 mm diameter, hydrophilic polytetrafluoroethylene (PTFE), 0.4 μm pore size). The use of porous Millicell inserts lifts the tissue slice off the bottom of the culture plate and holds the tissue slice at a fixed depth within the culture well. This helps to maintain a consistent air liquid interface (ALI). System C) Utilises the CELLBLOKS ^®^ cell culture system. This culture plate has 4 columns of 3 inter-linked barrier blocks that enable a gravity driven bidirectional flow of culture media by using a platform rocker.

Three culture systems were compared ( Figure 2). After recovery, 78 slices per patient were haphazardly transferred using a sterilised microspatula into: (A) 24-well plates without inserts, (B) Millicell standing inserts (Millipore, #PICM01250, PTFE CAS 9002-84-0), or (C) Revivocell CELLBLOKS ^®^ (RC). This resulted in an unbiased distribution ensuring slice variability was evenly distributed across systems. In conditions A and B, each well contained 450 μl supplemented WEM plus human epidermal growth factor (EGF) (Corning, #354052, CAS 62253-63-8) at 20 ng/mL ( Table 2), placed on an orbital shaker at 95 rpm. RC wells received 2.5 ml supplemented WEM, placed on a rocking platform. Media was changed on day 1 and every 48 hours.

Six slices per timepoint (Days 0, 3, 7, 11, 15) were used to assess viability and provide tissue for downstream analyses, addressing intra-patient variation. A power calculation ^12^ using variance estimates from Bigaeva, Gore ^13^ indicated n = 5 biological replicates (patients) to account for inter-patient variability. Because RC share media among three wells, we also pooled media from the corresponding slices in conditions A and B at each timepoint (D3, 7, 11, 15). This ensured a consistent approach across all culture systems, providing a single representative media sample from each. The pooled samples were then snap-frozen in liquid nitrogen (LN _2_) for future analysis.

### MTS viability assay

hPCTS viability was measured at selected timepoints (D0, 3, 7, 11, 15) using the CellTiter 96 ^®^ Aqueous One Solution Cell Proliferation Assay (Promega, #G3582, CAS 145315-52-2) (MTS). At each timepoint, six slices were removed from culture and placed into fresh 24-well plates (no insert) containing 400 μl supplemented WEM (without hEGF) and 80 μl MTS. Plates were incubated for three hours at 37°C, 5% CO _2_ on an orbital shaker, protected from light. After incubation, 200 μl of the MTS/WEM solution was transferred to a 96-well plate (STARLAB, #CC7682-7596) for absorbance measurement at 490 nm on a microplate reader (Varioskan Flash, Thermo Fisher, #VLBL00GD2). The same slices were then divided for histology,10% neutral buffered formalin (Thermo Fisher, #23-245684, CAS 50-00-0), or snap-freezing for additional analyses.

### Albumin and urea quantification

To evaluate slice functionality, albumin and urea production were assessed. Representative media samples were collected at D3, 7, 15 for each culture system. Urea was measured via a colorimetric Urea Assay kit (ab83362, Abcam, UK), with media diluted 1:50 in Assay Buffer (minimum 2 μl). Albumin was measured using a Human Albumin ELISA kit (ab108788, Abcam, UK), with media diluted 1:2 in dH _2_O (minimum 20 μl). Analyses followed each manufacturer’s protocol.

### Embedding/histology

Tissue slices were fixed in 10% neutral buffered formalin for a minimum of 24 hours. Specimens were then pre-processed using Liverpool Shared Research Histology facilities, dehydrated with 70%, 90% and 100% ethanol (CAS 64-17-5), cleared with 100% xylene (Fisher Scientific, X/0250/15, CAS 1330-20-7), impregnated and then embedded flat to facilitate transverse sectioning in paraffin wax (Merck, #76242-1KG). Wax blocks were sectioned at 4 μm using a microtome (Reichert-Jung 2030) and stained with haematoxylin (Surgipath, 3801540E, CAS #517-28-2) and eosin (Surgipath, 3801600E, CAS #17372-87-1). Sections were mounted with DPX (MilliporeSigma Cat #06522). The proportion of viable liver cells was confirmed following a subjective assessment by a blinded clinical histopathologist at 20x magnification on brightfield microscopy.

### Statistical analysis

GraphPad Prism 10.0.3 software was used for data visualisation and statistical analysis. ^14^ R studio with relevant packages (ggplot2, tidyverse, rstatix) can perform equivalent functions. ^15^ The mean of the six technical replicates was calculated for each viability data point, and media was pooled prior to testing for secretion analysis. Missing values were present in the RC datasets due to tissue slice infections, resulting in the exclusion of specific slices. Data were normalised by calculating the percentage change from Day 0 for tissue slice viability and from Day 3 for urea and albumin secretion for each culture method, allowing for comparison between patients. Data were assessed for normality using the Shapiro-Wilk test prior to multiple comparisons analysis. For normally distributed data with no missing values, repeated measures ANOVA (RM ANOVA) were performed to compare the viability data at each timepoint back to the Day 0 control, with Dunnett’s multiple comparisons post hoc analysis. For normally distributed data with missing values, a mixed-effects model was employed, followed by a Dunnett’s multiple comparisons test. Statistical significance was considered at P < 0.05 for both viability and media secretion data (urea and albumin).

## Results

### Viability

**Figure 3.**
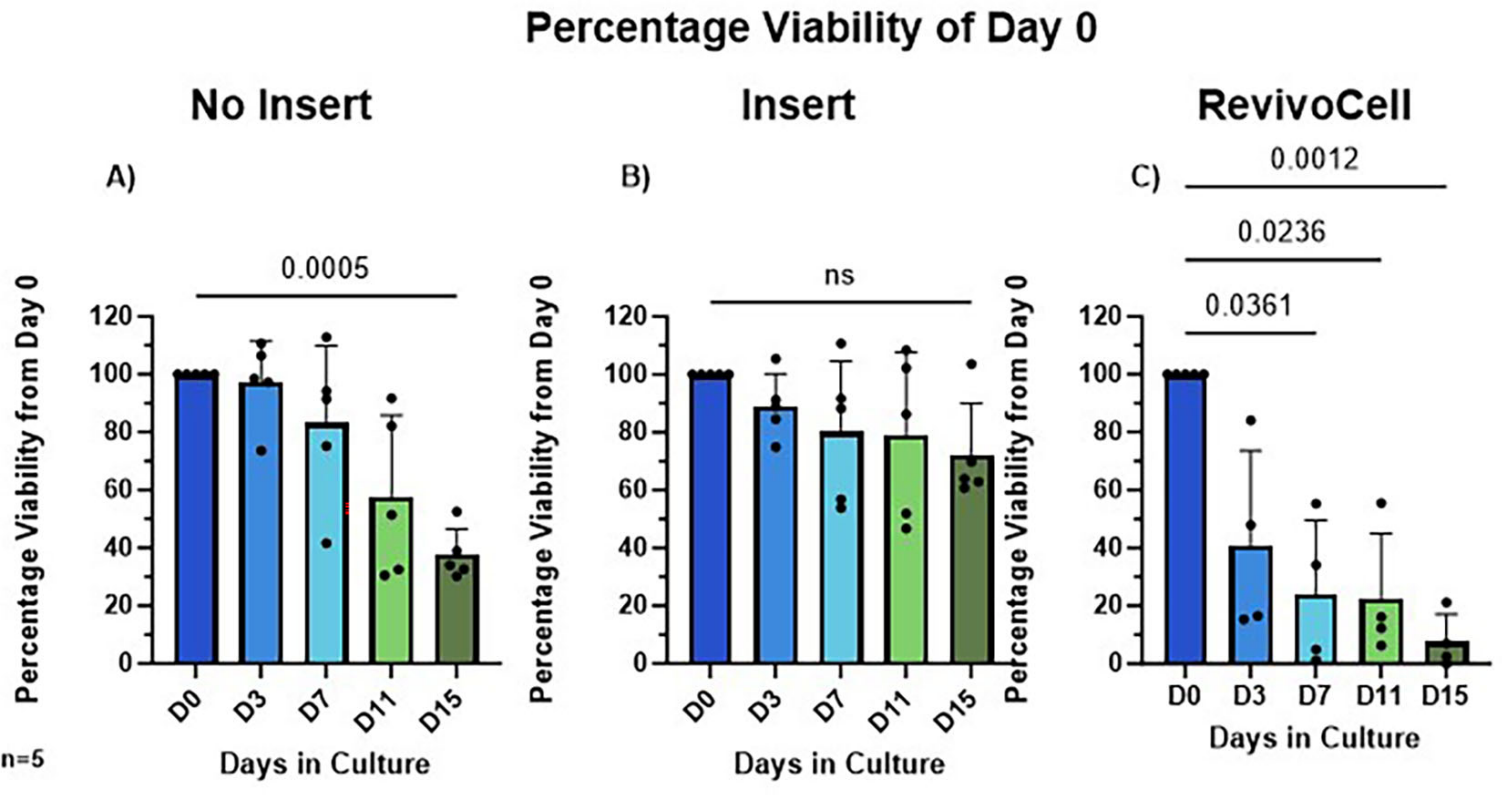
Percentage viability change over 15 days of three different methods of liver hPCTS culture. Viability measured using MTS assay, absorbance measured at 490 nm and the percentage of Day 0 Viability calculated (A) No insert culture (B) Insert culture (C) Revivo Cell culture. Mean ± SD, Mixed-Effects Analysis, Dunnett’s multiple comparison post hoc test. P < 0.05. n = 5 patients.

To establish the best method for long-lived hPCTS cultures, we compared three different culture systems and assessed viability over 15 days ( Figure 3). Statistical analysis was conducted using mixed-effects analysis followed by Dunnett’s post-hoc test for multiple comparisons. The mean MTS absorbance across all patients at day 0 was 2.135 (95% CI 1.97-2.30, SD 0.44). For the no insert system, a significant decline in viability relative to Day 0 was observed by Day 15 (D0 vs. D15: 100% vs. 37.69%, p = 0.0005, Mean Diff. (MD) 62.31, SD = 8.906, 95% CI: [43.87, 80.75]). In contrast, the Millicell insert system showed no significant change in viability over the 15 days (D0 vs. D15: 100% vs. 72.23%, p = 0.1227, SD = 17.87, MD 27.77, 95% CI: [-9.24, 64.78]). For the RC system, significant reductions in viability were seen much earlier and observed at Day 7 (D0 vs. D7: 100% vs. 23.94%, p = 0.0361, SD = 25.59, MD 76.06, 95% CI: [8.53, 143.60]), Day 11 (D0 vs. D11: 100% vs. 22.60%, p = 0.0236, SD = 22.37, MD 77.40, 95% CI: [18.38, 136.4]), and Day 15 (D0 vs. D15: 100% vs. 7.67%, p = 0.0012, SD = 9.504, MD 92.33, 95% CI: [67.25, 117.40]).

### Function

**Figure 4.**
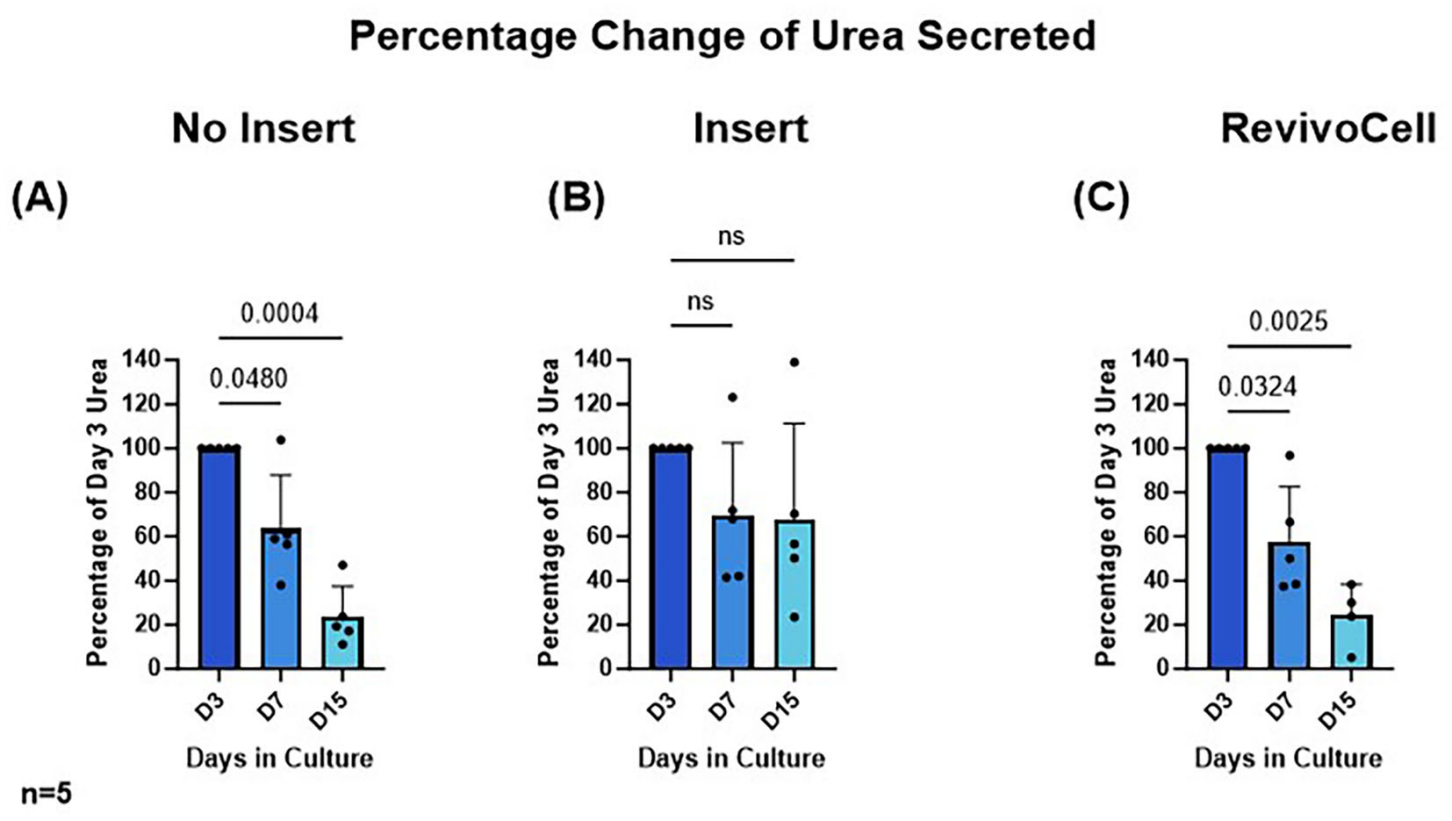
Change in urea secreted into culture media over 15 days. HPCTS cell culture media collected at different time points and underwent urea ELISA. Days 7 and 15 were calculated as a percentage of urea levels identified after 3 days in culture A) NI = No insert culture (B) I = Insert culture (C) RC = Revivocell culture. Mean ± SD, RM One-way ANOVA (NI, I), Mixed-Effects Analysis (RC), Dunnett’s multiple comparison post hoc test. P < 0.05. n = 5 patients. Mean urea values at day 3 for NI, I and RC were 5.623 (SD, 2.294), 3.124 (SD, 0.9843), and 3.192 (SD, 0.7901) nmol/μl respectively.

**Figure 5.**
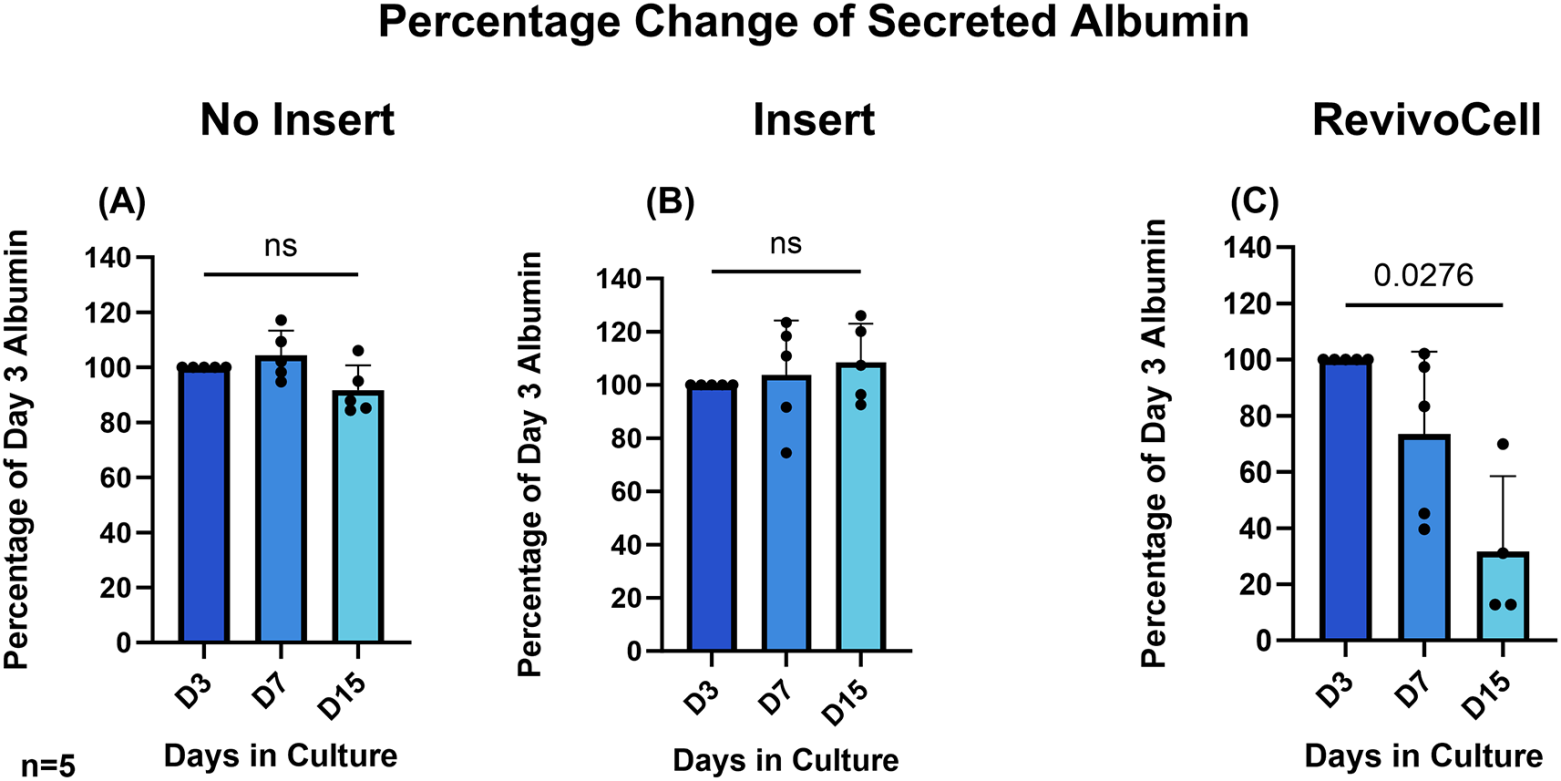
Change in albumin secreted into culture media over 15 days. HPCTS cell culture media collected at different time points and underwent albumin ELISA. Days 7 and 15 were calculated as a percentage of albumin levels identified after 3 days in culture A) NI = No insert culture (B) I = Insert culture (C) RC = Revivocell culture. Mean ± SD, RM One-way ANOVA (NI, I), Mixed-Effects Analysis (RC), Dunnett’s multiple comparison post hoc test. P < 0.05. n = 5 patients. Mean Albumin values at day 3 for NI, I and RC were 540.4 (SD, 52.05), 514.9 (SD, 79.29), and 517.9 (SD, 34.69) ng/ml respectively.

To determine hPCTS functionality we assessed hepatic albumin and urea production by identifying these excreted proteins within the culture media at D3, D7 and D15. Due to the frequency of media changes this ensured a consistent 48-hour period in which both secreted albumin and urea could accumulate in the culture media. As a result, albumin and urea production were calculated as a percentage change relative to day 3 levels. These data showed that albumin and urea levels were most stable in hPCTS cultured on inserts (system B) ( Figure 4 & Figure 5). Whilst albumin production was also relatively stable in hPCTS cultured without inserts (system A), analysis revealed a significant decrease in urea production in these slices from day 3 to 7 (D3 vs. D7: 100% vs. 63.60%, p = 0.0480, SD = 24.26, MD 36.40, 95% CI: [0.4843, 72.32]) and day 3 to 15 (D3 vs. D15: 100% vs. 23.69%, p = 0.0004, SD = 13.85, MD 76.31, 95% CI: [55.80, 96.83]) ( Figure 4).

There was a significant reduction in relative levels of albumin (D3 vs. D15: 100% vs. 31.63%, p = 0.0276, SD = 26.92, MD 68.37, 95% CI: [13.62, 123.1]) and urea (D3 vs. D7: 100% vs. 57.84%, p = 0.0324, SD = 24.76, MD 42.16, 95% CI: [5.443, 78.88]) (D3 vs. D15: 100% vs. 24.35%, p = 0.0025, SD = 14.11, MD 75.65, 95% CI: [49.69, 101.6]) produced by hPCTS cultured on the RC system ( Figure 5). This was not unexpected and correlated with the significant reduction in hPCTS viability seen in when using this culture system.

### Histology

**Figure 6.**
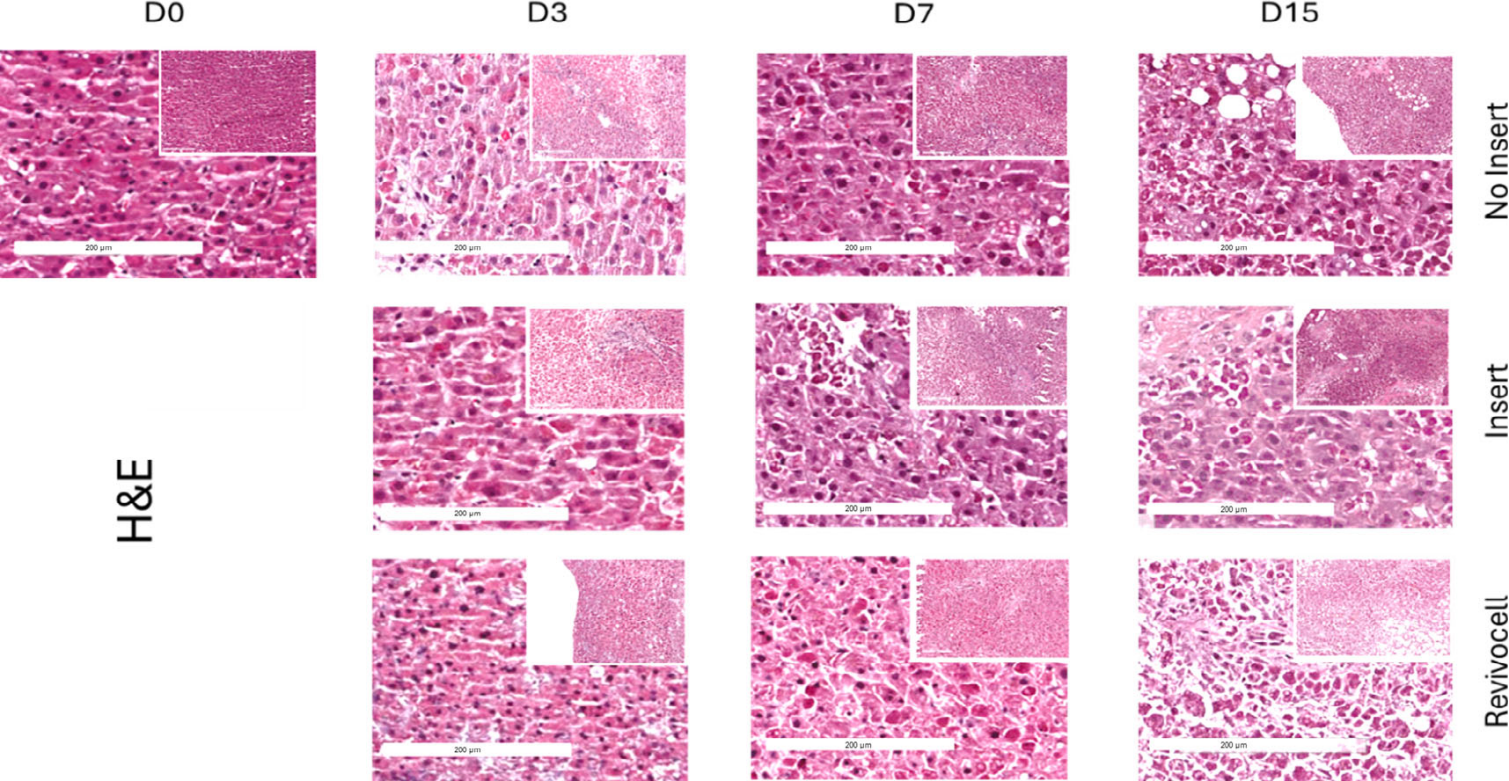
Representative H&E-stained images of human precision-cut tumour slices (hPCTS) over 15 days in culture, comparing No Insert, Insert, and Revivocell culture methods. All images were acquired at 20× magnification using a Leica Aperio CS slide scanner, highlighting morphological changes and tissue integrity under each condition.

To determine the histological integrity of our hPCTS cultures and assess for maintenance of tissue architecture, H&E slides were blindly examined by a clinical histopathologist from RLUH. To ensure blinding, slides from each patient and timepoint (Days 0, 7, and 15) were provided without identifiers for culture system, timepoint, or patient. The histopathologist then assessed and subjectively graded the slides based on the proportion of viable liver cells across the three culture systems. Insert cultured slides were deemed to have the highest proportion of viable liver cells and best retention of cellular architecture when examined at x20 magnification ( Figure 6).

Long-lived cultures are essential for maximising the clinical utility of hPCTS and for realising the potential of this platform as a preclinical model. We show that by using Millicell inserts tissue slices derived from human liver can be cultured ex-vivo and maintain viability and functionality for up to 15 days. These findings mirror those reported by Wu *et al.* who also showed that MTS activity in hPCTS cultured on trans-well inserts could be sustained to D15. ^16^ In contrast, hPCTS cultured without a supportive insert show a significant reduction in viability relative to D0, with D15 slices essentially non-viable on MTS assessment.

The number of variations (inserts, flow systems, media composition, slicing techniques, viability assessments) in tissue slice culture and the bespoke nature of some of these systems can make direct comparison between published results difficult when trying to determine optimal conditions. Paish *et al.* developed a culture system (BioR plate) consisting of hPCTS cultured on trans-well inserts in wells connected by a flow channel and showed this to further improve hPCTS viability and functionality. ^7^ We sought to replicate this by using a commercially available culture plate that combines organotypic inserts within interlinked culture blocks (RC). Unlike Paish *et al.*, we found that hPCTS cultured on a flow system to perform poorly with significantly worse survival. This correlated with a significant reduction in relative albumin and urea secretion into culture media. In contrast, hPCTS cultures on Millicell inserts had stable albumin and urea secretion with an absolute albumin production (ng/ml) similar to that described in other studies. ^7, 17^

A significant issue encountered with the RC system was the slices at the end of each row were lifting off their inserts as the platform rocked. On occasion the slices would be then deposited back on the plastic surrounds of the insert. Altering the speed and intensity at which the platform rocked failed to ameliorate this problem. To compensate for this issue, we reduced the media volume from the recommended 3 ml to 2.5 ml. Whilst this solved the issue of the slices being washed off the insert it may have affected the flow properties of the plate resulting in suboptimal movement of media between the wells, affecting viability. We acknowledge that the RC plate was not designed for use with tissue slices but rather primary cell culture with the purpose of facilitating co-culture and cross talk between different cell populations. ^18^ Another potential variable affecting viability is structure of the insert. Millicell Organotypic Biopore™ Inserts are constructed using polytetrafluoroethylene (PTFE) that is extruded and rolled to form a interconnected semi-porus fibrous membrane with 0.4 μm pores. The inserts within the RC plate are created from a polyethylene terephthalate (PET) membrane that is then bombarded with energetic particles to produce near identical 0.4 μm pores. Although both inserts have the same pore size, the membrane structure is vastly different. Other studies have attributed PFTE having a more organotypic barrier compared to PET membranes. ^19, 20^

Overall, we found that the Millicell organotypic insert was the most user friendly method for maintaining hPCTS in culture and demonstrated the most consistent viability and secretion of albumin and urea over the 15 days. The use of RC plate has shown promising developments in cell line culture, however when combined PCTS the same advantages could not be replicated.

## Ethics and consent

Human liver tissue was obtained by qualified medical staff at The Royal Liverpool University Hospital (Liverpool, UK). Written, informed consent was obtained from each patient. Ethical approval and written consent were obtained under the NIHR PINCER platform study (NW REC 15/NW/0477), ^8^ and the University of Liverpool Ethics Board adhering to the declaration of Helsinki. ^9, 10^ All demographic data were anonymised.

## Data availability

### Underlying data

Zenodo: “Comparing methods of human precision cut tissue slice culture - tissue slice viability and functional markers data” DOI: 10.5281/zenodo.15089327. ^21^

This project contains the following underlying data:

• **Viability Data NC3Rs paper.xlsx** – MTS viability data for human precision cut tissue slices (hPCTS) cultured over 15 days using three different culture methods (no insert, Millicell insert, CELLBLOKS ^®^).
(File size: ~51 kB; MIME type: application/vnd.openxmlformats-officedocument.spreadsheetml.sheet)
• **Urea and Albumin Data NC3Rs paper.xlsx** – Percentage change in urea and albumin secretion into media from Day 3 in hPCTS cultured over 15 days.
(File size: ~19 kB; MIME type: application/vnd.openxmlformats-officedocument.spreadsheetml.sheet)
• **README file** – A comprehensive file detailing the dataset, including general information, methodology, file descriptions, and additional metadata.

The dataset is provided under a CC BY 4.0 ( Creative Commons: Attribution-Noncommercial-Share Alike 4.0) license

### Extended data

Zenodo: “Comparing methods of human precision cut tissue slice culture - tissue slice viability and functional markers data” DOI: 10.5281/zenodo.15089327. ^21^

This project contains the following underlying data:

• **Extended data consumables list.docx** – A list of consumables required for this brief report.

The dataset is provided under a CC BY 4.0 ( Creative Commons: Attribution-Noncommercial-Share Alike 4.0) license.
