# Supplementary material for: A Preclinical Model of Human Liver Using Precision Cut Tissue Slice Culture
Source: F1000Res. 2025 Jun 10;14:571. [Version 1] doi: 10.12688/f1000research.162495.1 (PMC12329406; doi:10.12688/f1000research.162495.1)
Supplement: Peer review history

# Reviewer report: Reviewer response for version 1

**DOI:** 10.5256/f1000research.178704.r391048

**Published:** 2025-08-13

**Authors:** Una Rastovic, Elena Palma

**Affiliations:** 1 Roger Williams Institute of Liver Studies, King's College London School of Immunology & Microbial Sciences, London, England, UK

In the study titled “A Preclinical Model of Human Liver Using Precision-Cut Tissue Slice Culture,” Owen McGreevy and colleagues compare three different culture systems for maintaining human precision-cut liver slices (PCLS) over a 15-day period. Specifically, they evaluate traditional 24-well plates, 24-well plates with polytetrafluoroethylene (PTFE) Millicell® inserts, and the CELLBLOKS® hydrophilic polyethylene terephthalate (PET) flow system. To assess liver slice viability and function, the authors measured metabolic activity using the MTS assay and quantified urea and albumin levels in culture supernatants. These functional assays were further supported by histological evaluation performed by a liver histopathologist. The authors conclude that the PTFE Millicell® insert system supports superior long-term maintenance of PCLS viability and functionality compared to the other two systems tested.

 The manuscript addresses a highly relevant and timely objective: prolonging the viability of human PCLS cultures, and is generally well structured and clearly written. The effort to compare multiple culturing systems using human-derived tissue is especially valuable, and the study has the potential to contribute meaningfully to the optimisation of long-term liver slice culture protocols.

 That said, there are several important methodological and analytical issues that currently limit the strength and reproducibility of the conclusions. In particular, concerns related to data normalisation, statistical testing, and clarity of presentation need to be addressed to ensure that the findings are robust and the comparative performance of the culture systems is fully interpretable.

 With careful major revision and attention to the points outlined below, this work could make an informative contribution to the field.

 Major comments:

1. In quantifying MTS, urea and albumin secretion in Figures 3, 4 and 5, the authors used normalised data in which the control group (D0 or D3) was set to 100%, resulting in zero variance within that group. This violates key assumptions of one-way ANOVA, including homogeneity of variances and the ability to estimate within-group error. Therefore, one-way ANOVA is not appropriate for this analysis. Ideally, the authors should have analysed the raw (non-normalised) data using one-way ANOVA or a mixed-effects approach that models repeated measures (i.e. patient-matching), or select a nonparametric test if using normalised data, but only if repeated measurements are clear and balanced.
2. Timing and comparability of Day 0 MTS measurements (Figure 3): It is unclear when the Day 0 (D0) samples for the MTS assay were collected and assessed. Are the D0 values identical across all conditions in Figure 3, such that direct comparisons of viability between the different culture systems are valid? Please clarify whether the D0 reference values were measured separately for each condition or pooled from a single baseline measurement—and whether this was done before or after the recovery period.
3. The authors report albumin and urea secretion data starting from Day 3, with values normalised to Day 3 (set at 100%) within each condition. They state that the 48-hour media change interval ensures consistent metabolite accumulation; however, it is unclear whether this assumption was validated during the early phase of culture, when tissue adaptation to different systems may affect functionality and potentially confound interpretation of later timepoints. Specifically, were albumin and urea levels measured at the first media change (e.g., Day 1), and were these analytes detectable then? Including Day 1 data and showing absolute (raw) secretion values would provide critical insight into early culture performance and strengthen conclusions about the relative efficacy of the three systems. Moreover, normalising to Day 3 values complicates direct comparisons between culture systems, since slices had already been cultured under different conditions for three days before this baseline. Therefore it would be valuable to understand the performance of the three systems at the first medium change too (including Day 1). Presenting raw data across all three conditions at multiple timepoints (as opposed to presenting longitudinal data for different conditions), would allow better assessment of each system’s performance. This is particularly important for comparing insert and no-insert conditions, which were cultured in identical medium volumes and could be directly contrasted.
4. Please clarify the reason for the absence of Day 11 data from Figures 4 and 5.
5. Could the authors clarify the apparent discrepancy between MTS results in Figure 3, which suggest non-viable slices, and the detectable albumin and urea secretion data in Figures 4 and 5 that indicate ongoing metabolic activity?
6. In the introduction, the authors state: “De Graaf et al. (2010) published a standardised liver and intestinal hPCTS protocol, renewing interest in this technique.5 This was limited by relatively short-term viability of several days due to issues maintaining sufficient oxygenation and nutrition. To extend viability, inserts have been used to help maintain an air-liquid interface producing a physiologically relevant oxygen concentration gradient.6” However, reference 6 is based on mathematical modelling and does not provide experimental evidence supporting the claim that inserts improve air-liquid interface maintenance or extend viability. The authors should either remove this reference or adjust the claim accordingly. Furthermore, key studies reporting human precision-cut liver slice cultures beyond 72 hours are not cited, nor are important references addressing strategies to overcome oxygenation and nutrient limitations through media composition and high oxygen atmospheres. A more comprehensive literature review is needed to clearly demonstrate the relevance of the current work.

In addition, the authors should consider addressing the following minor points to improve clarity and rigor of the manuscript.

 Minor comments:

1. In human-derived models, donor variability is often a strong influencing factor, which is also apparent in the current data. Moreover, the quality of the starting material appears to be a key determinant of culture success, highlighting the need for rigorous screening of tissue quality and viability prior to culture. Were there any baseline donor-specific differences in MTS values at Day 0 that might indicate variability in tissue quality? Were any cultures excluded due to poor viability at baseline? Please include details.
2. Did the authors observe any macroscopic differences between the culture systems, such as slice curling, damage to surface edges, or changes in slice weight over time? Please comment.
3. Table 2 currently lists only the medium composition. Please also include the composition of the Krebs-Henseleit Buffer (KHB) used, along with details regarding its oxygenation (if applicable) and how frequently was replaced during slicing.
4. How was the slice thickness assessed during slice preparation? Please provide details.
5. For better comparison with other human PCLS studies, it would be helpful to include a schematic timeline illustrating the medium change schedule and how the timepoints relate to the day of surgery. Inclusion of such a timeline would aid reader understanding.
6. The inclusion of the statement about R Studio and associated packages (ggplot2, tidyverse, rstatix) appears unrelated to the main text. Please clarify or consider removing it.
7. Please note that urea is a small molecule (carbamide) rather than a protein. Kindly correct this in the manuscript.
8. For the viability assessment, the authors state that the mean of six technical replicates was calculated for each data point. Was there variability between slices from the same donor and were any outliers identified and excluded? Please provide details.
9. Please clarify whether rocking or orbital shaking was used for the RC system during culture.
10. Kindly specify the type of incubator used, including details on the gas composition during culture.
11.  An inherent feature of precision-cut liver slices is the induction of a wound healing response due to mechanical injury during slice preparation. In addition to assessing viability and functionality, have the authors evaluated any markers of wound healing or hepatic stellate cell activation, such as collagen expression? Including such analyses would provide valuable insight into how different culture systems influence tissue repair processes, particularly in prolonged cultures of up to 15 days.

Is the work clearly and accurately presented and does it cite the current literature?

Partly

If applicable, is the statistical analysis and its interpretation appropriate?

No

Are all the source data underlying the results available to ensure full reproducibility?

Partly

Is the study design appropriate and is the work technically sound?

Yes

Are the conclusions drawn adequately supported by the results?

No

Are sufficient details of methods and analysis provided to allow replication by others?

Partly

Reviewer Expertise:

Patient-Derived Liver Disease Models · Precision-Cut Liver Slices · Ex Vivo Tissue Function & Viability · ECM Remodelling & Fibrosis · Mitochondrial Dynamics & Morphology

We confirm that we have read this submission and believe that we have an appropriate level of expertise to confirm that it is of an acceptable scientific standard, however we have significant reservations, as outlined above.

# Reviewer report: Reviewer response for version 1

**DOI:** 10.5256/f1000research.178704.r391049

**Published:** 2025-08-06

**Authors:** Sonam Gurung

**Affiliations:** 1 University College London, London, England, UK

The authors present a comprehensive protocol for culturing human precision-cut tissue slices (hPCTS). This platform preserves the native three-dimensional architecture and microenvironment of human tissues, thereby enhancing physiological relevance. Such an approach offers significant advantages for therapeutic screening and disease modelling, aligning well with the principles of the 3Rs (Replacement, Reduction, and Refinement) in animal research. The methodology also shows potential for application to a range of other tissue types.

 A major limitation of similar models has been their short-term viability. In this study, the authors demonstrate that their system maintains tissue viability for up to 15 days, with sustained levels of albumin and urea secretion and preserved histological architecture.

**Questions for the authors:**

1. The liver biopsies were derived from patients. Was there any observed correlation between the patient's clinical condition and the health or viability of the liver tissue slices over time?
2. Is there a defined time window between tissue harvest and culture initiation that is critical for maintaining optimal slice viability?
3. There appears to be some inconsistency in terminology particularly in the figure legends—some refer to “human precision tumor slices,” while others use the full term “human precision tissue slices” (hPCTS). Can this be clarified for consistency?
4. Statistical comparisons are made only against Day 0 for MTS. Should we infer that differences between other timepoints were not statistically significant? This also applies to the urea and albumin measurements.
5. The observed decline in urea and albumin secretion over time—especially in the Revivocell condition—may just be due to decreased cell viability at later timepoints. Could reduced cell numbers be accounted for in the analysis.
6. For the histological assessment, how many images per tissue slice were captured and analyzed?

Is the work clearly and accurately presented and does it cite the current literature?

Yes

If applicable, is the statistical analysis and its interpretation appropriate?

I cannot comment. A qualified statistician is required.

Are all the source data underlying the results available to ensure full reproducibility?

No source data required

Is the study design appropriate and is the work technically sound?

Yes

Are the conclusions drawn adequately supported by the results?

Yes

Are sufficient details of methods and analysis provided to allow replication by others?

Yes

Reviewer Expertise:

metabolic disorders, non-viral gene therapy, ex vivo models.

I confirm that I have read this submission and believe that I have an appropriate level of expertise to confirm that it is of an acceptable scientific standard.

# Reviewer report: Reviewer response for version 1

**DOI:** 10.5256/f1000research.178704.r391054

**Published:** 2025-07-29

**Authors:** Raymond S W Yeung

**Affiliations:** 1 University of Washington, Seattle, Washington, USA

This report by McGreevy et al. aimed to study methodologic parameters involved in organotypic liver slice culture as a model platform to investigate human livers. They found that the use of PFTE support membrane gave optimal results in their hands, which is consistent with prior reports. Surprisingly, introducing 'flow' using the CELLBLOKS system created less viability.  They authors attributed the effects to the use of a PET membrane, although that was not directly compared. Nonetheless, the findings help to confirm the utility of the tissue slice culture for studying human diseases and the technical details are useful to the scientific community.

Is the work clearly and accurately presented and does it cite the current literature?

Yes

If applicable, is the statistical analysis and its interpretation appropriate?

Yes

Are all the source data underlying the results available to ensure full reproducibility?

Yes

Is the study design appropriate and is the work technically sound?

Yes

Are the conclusions drawn adequately supported by the results?

Yes

Are sufficient details of methods and analysis provided to allow replication by others?

Yes

Reviewer Expertise:

Personalized oncology and human tumor response

I confirm that I have read this submission and believe that I have an appropriate level of expertise to confirm that it is of an acceptable scientific standard.
